# Cardamonin mitigates kidney injury by modulating inflammation, oxidative stress, and apoptotic signaling in rats subjected to renal ischemia and reperfusion

**DOI:** 10.25122/jml-2023-0093

**Published:** 2023-12

**Authors:** Haidar Hameed Ali Al-Sultany, Murooj Altimimi, Heider Qassam, Najah Rayish Hadi

**Affiliations:** 1 Department of Pharmacology and Therapeutics, Faculty of Medicine, University of Kufa, Najaf, Iraq

**Keywords:** renal ischemia, renoprotection, Cardamonin, blood urea nitrogen (BUN)

## Abstract

Renal ischemia-reperfusion injury (IRI) is a critical health concern that aggravates the pathophysiology of acute kidney injury (AKI), leading to high mortality rates in intensive care units. Cardamonin is a natural compound with anti-inflammatory and antioxidant properties. The current study aimed to evaluate the renoprotective impact of cardamonin against AKI induced by renal IRI. Male rats (n=5 per group) were divided into four groups: the sham group underwent anesthesia and abdominal incision only; the control group experienced bilateral renal artery clamping for 30 minutes followed by 2 hours of reperfusion; the vehicle group received the cardamonin vehicle 30 minutes before ischemia induction; and the cardamonin group was administered 5 mg/kg of cardamonin 30 minutes before ischemia. Blood urea nitrogen (BUN) and creatinine were measured to assess the renal function. Tumor necrosis factor alpha (TNF-α), interleukin 1 beta (IL-1β), interleukin-6 (IL-6), caspase 3, and F2-isoprostane were assessed in renal tissues. Kidney injury was examined using the hematoxylin and eosin stain method. Compared to the sham group, the control group exhibited significantly higher levels of BUN, creatinine, TNF-α, IL-1β, IL-6, F2-isoprostane, and caspase 3 in renal tissues, along with severe kidney injury as evidenced by histological analysis. Compared to the control group, pretreatment with cardamonin resulted in a significant reduction in these biomarkers and alleviated renal damage. Cardamonin had renoprotective effects against renal ischemia and reperfusion injury via modulating inflammation, oxidative stress, and apoptosis pathways.

## INTRODUCTION

Ischemia-reperfusion injury (IRI) is a clinical condition characterized by structural and functional changes that occur because of a sudden loss of blood supply followed by blood restoration. IRI involves two phases: the ischemic phase, marked by the loss of cellular energy, and the reperfusion phase, characterized by cellular events such as inflammation, oxidative stress, apoptosis, and necrosis [1]. A significant consequence of renal IRI is acute kidney injury (AKI), commonly observed in various clinical scenarios, including kidney transplantation, sepsis, burns, and vascular surgery. AKI affects more than thirteen million patients across the world, with IRI being a leading cause [2]. Despite advancements in AKI treatment in critical care units, challenges persist for clinicians, especially since AKI increases the risk of damage to multiple organs, including the lungs, heart, liver, and brain [3].

Following IRI, inflammatory-associated biomarkers such as nuclear factor kappa B (NF-κB), interleukin-6 (IL-6), interleukin-1 beta (IL-1β), and tumor necrosis factor-alpha (TNF-α) are activated in the kidneys, leading to tissue damage characterized by endothelial and tubular cell dysfunction [4]. Both IL-1β and TNF-α, key proinflammatory cytokines, are released in response to IRI and regulate and modulate inflammation [5]. During the reperfusion phase, there is an infiltration of neutrophil cells, excessive production of TNF-α and IL-1β, and activation of NF-κB in renal tissues, suggesting a link between these factors in mediating IRI [6]. Oxidative stress and inflammation are key players in deteriorating kidney tissues, leading to apoptosis and necrosis [7, 8].

Cardamonin (CDM), derived from the *Zingiberaceae* family, has gained attention for its multi-target properties, particularly its antioxidant, anti-inflammatory, and antiapoptotic effects [9, 10]. It has been reported that CDM has protective properties via its impact on various signaling pathways involved in different clinical conditions such as cancer, diabetes, cardiovascular diseases, and inflammatory diseases [11, 12]. This study aimed to investigate the renoprotective potential of CDM against AKI induced by IRI.

## MATERIAL AND METHODS

### Animal handling and care

All experimental procedures were performed at the laboratories of the Faculty of Medicine, University of Kufa, Iraq. Animals were obtained from The National Centre for Control and Pharmaceutical Research, Ministry of Health, Iraq. Animals were maintained under standardized conditions (25 °C, 60-65 humidity, 12 light/12 dark cycles) and allowed access to free water and food before the experiments. All experiments were subjected to strict guidelines and granted by the Animal Care and Use Committee.

### Study design

Twenty Sprague Dawley rats (200-350 g, aged 20-24 weeks) were randomly divided into four groups (n=5 each): the sham group received anesthesia and underwent only abdominal opening; the control group underwent anesthesia, abdominal incision, and renal ischemia induced by clamping the renal arteries for 30 minutes followed by 2 hours of reperfusion [13, 14]; the vehicle-treated group received the cardamonin vehicle intraperitoneally (i.p.) (10% DMSO, 40% PEG300, 5% Tween 80, and 45% normal saline) 30 minutes before ischemia; the cardamonin group received 5 mg/kg of cardamonin i.p. 30 minutes before ischemia [15, 16].

### Renal ischemia and reperfusion injury procedure

The animals were anesthetized with ketamine (100 mg/kg, i.p) and xylazine (10 mg/kg, i.p) as previously described [17]. A midline incision was made, and renal pedicles were exposed and clamped for 30 min using vascular clamps [18]. After the ischemic period, the clamps were released for 2 hours of reperfusion [19]. The abdomen was then closed, and the animals were rehydrated with an i.p. injection of normal saline (1 ml). At the end of the experiment, the animals were euthanized under deep anesthesia, and blood was collected from the heart to measure BUN and creatinine levels. The left kidney was put in 10 % formaldehyde for histological examination, and the right kidney was stored at -80 °C to measure the levels of bioindicators in renal tissues.

### Cardamonin preparation

Cardamonin (Pub Chem CID: 641785) was obtained as a pure powder from MedChemExpress, USA. Cardamonin was dissolved in a solvent containing 10% DMSO, 40% PEG300, 5% Tween-80, and 45% normal saline. Cardamonin was administered at 5 mg/kg intraperitoneally [20].

### Renal function test

Blood (~3.5 ml) was collected from the heart, allowed to stand for 30 minutes at room temperature in an anticoagulant-free tube, and then centrifuged at 3000 rpm for 10 minutes at 4°C. The serum was used to measure blood urea nitrogen (BUN) and creatinine levels using commercial kits.

### Enzyme-linked immunosorbent assay

Kidneys were removed and washed with ice-cold normal saline. To make tissue homogenates, the kidney was weighted and put in a phosphate buffer saline containing 1 % Triton X-100 and a protease inhibitor in a ratio of 1 to 10 (w/v). Samples were processed by ultrasonic processer at 4 °C and centrifuged at 14000 rpm for 15 minutes. The supernatant was used to measure TNF-α, IL-1β, IL-6, caspase 3, and F2-isoprostane levels.

### Hematoxylin and eosin staining

Kidneys were fixed in 10% formaldehyde, embedded in paraffin, and sectioned horizontally at 5 micrometers. Sections were stained with hematoxylin and eosin (H&E) and examined under a light microscope by two independent investigators blinded to the study groups. Renal tissue damage was scored as follows: 0, no damage; 1, ≤25% tubular injury; 2, 25-50% tubular injury; 3, 50-75% tubular injury; 4, >75% tubular injury. Features of renal injury included the loss of brush borders, epithelial swelling, cytoplasmic eosinophilia, vacuolar degeneration, eosinophilic cast formation, and necrosis [21].

### Data analysis

Data were analyzed and represented using GraphPad Prism version 7. Results are presented as mean ± standard error of mean unless otherwise stated. One-way analysis of variance (ANOVA) followed by Tukey's test was used for comparisons among groups. For non-parametric data, which included scores of renal damages, the Kruskal Wallis test was used, followed by the Tukey post hoc test. Levels of significance were considered at a p-value of 0.05.

## RESULTS

### Impact of CDM on renal function indicators

**Figure 1:**
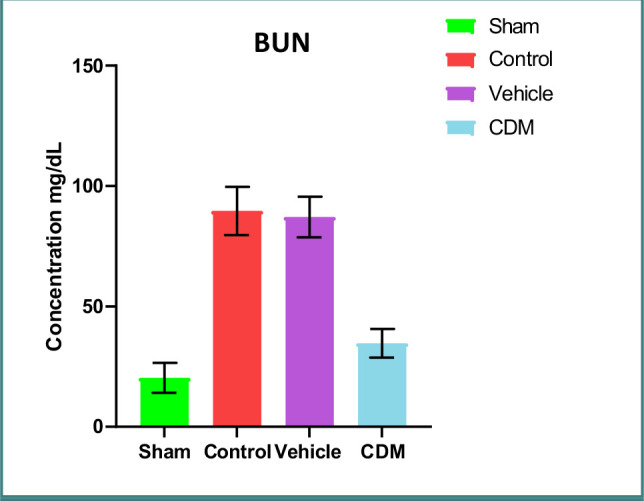
Effect of CDM on BUN levels following renal IRI Serum BUN (mg/dl) across groups. Mean±SEM, n=5. *P≤0.05 vs sham; #P≤0.05 vs control/vehicle.

**Figure 2:**
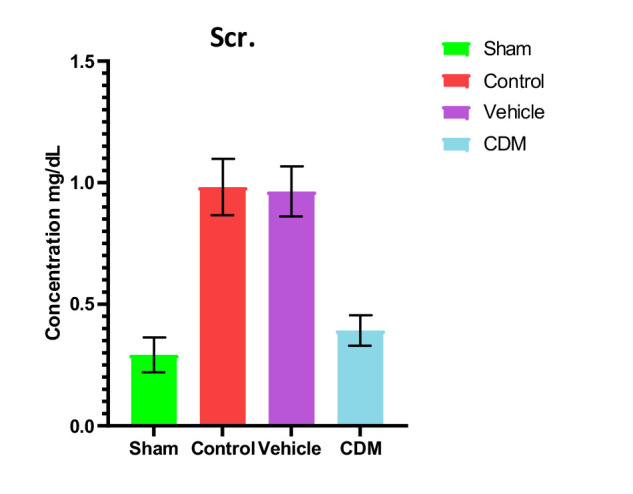
Effect of CDM on creatinine levels following renal IRI Serum creatinine (mg/dl) across groups. Mean±SEM, n=5. *P≤0.05 vs sham; #P≤0.05 vs control/vehicle.

The serum levels of BUN and creatinine were significantly higher in the control and vehicle groups compared to the sham group (Figures 1 and 2). Treatment with CDM reduced these levels compared to the control group (Figures 1 and 2).

### Impact of CDM treatment on oxidative stress in the renal tissue

**Figure 3:**
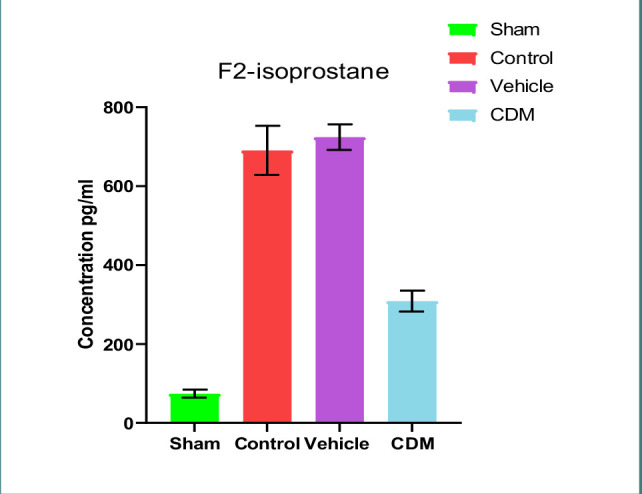
Effect of CDM on F2-isoprostane levels following renal IRI F2-isoprostane levels (pg/ml) across groups. Mean±SEM, n=5. *P≤0.05 vs sham; #P≤0.05 vs control/vehicle.

The levels of F2-isoprostane, a bioindicator of oxidative stress, were significantly elevated in the control and vehicle groups compared to the sham group (Figure 3). In contrast, pretreatment with CDM resulted in a significant reduction in the levels of F2-isoprostane compared to the control and vehicle groups (Figure 3).

### Impact of CDM treatment on TNF-α and IL-1β in the renal tissues

**Figure 4:**
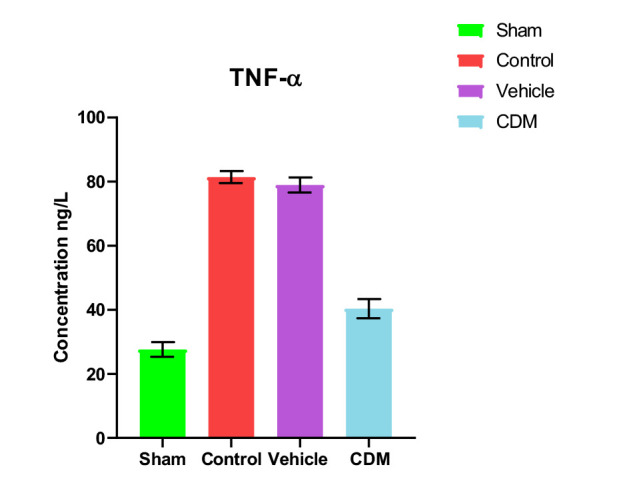
Effect of CDM on TNF-α levels following renal IRI TNF-α levels (ng/l) across groups. Mean±SEM, n=5. *P≤0.05 vs sham; #P≤0.05 vs control/vehicle.

**Figure 5:**
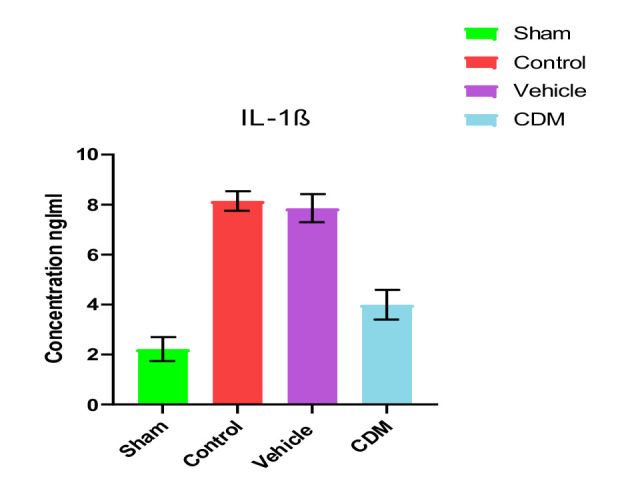
Effect of CDM on IL-1β following renal IRI IL-1β levels (ng/ml) across groups. Mean±SEM, n=5. *P≤0.05 vs sham; #P≤0.05 vs control/vehicle.

Data showed increased levels of TNF-α and IL-1β in the renal tissues of rats in control and vehicle groups compared to the sham group (Figures 4 and 5). Compared to the control and vehicle groups, these levels were significantly reduced when rats were pretreated with CDM (Figures 4 and 5).

### Impact of CDM treatment on caspase 3

**Figure 6:**
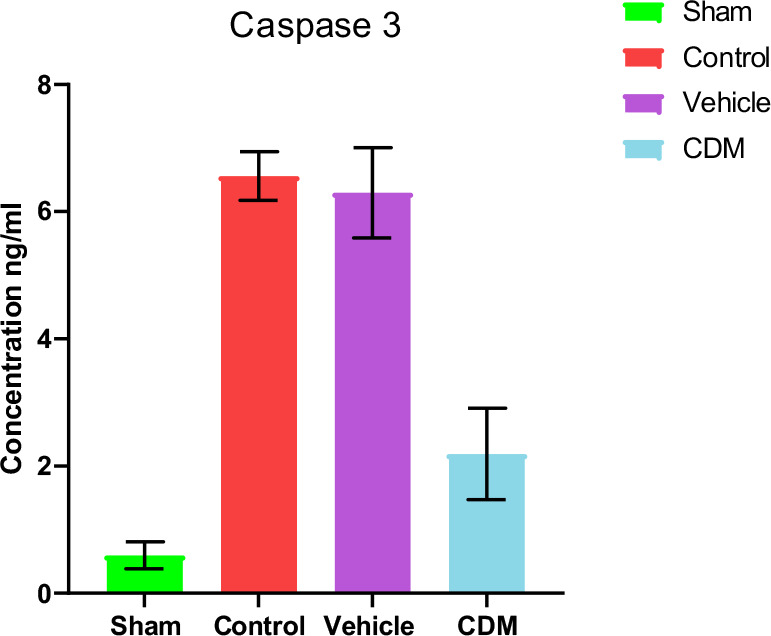
Effect of CDM on caspase 3 following renal IRI Caspase 3 levels (ng/ml) across groups. Mean ± SEM, n=5. *P≤0.05 vs sham; #P≤0.05 vs control/vehicle.

The levels of caspase 3 in the renal tissues were assessed to explore the potential effect of CDM on apoptosis. The findings showed that caspase 3 levels were significantly higher in the control and vehicle groups compared to the sham group (Figure 6). In contrast, pretreatment with CDM led to a significant decrease in caspase 3 levels in the renal tissues, indicating a significant reduction in apoptosis (Figure 6).

### Histopathological findings

**Figure 7:**
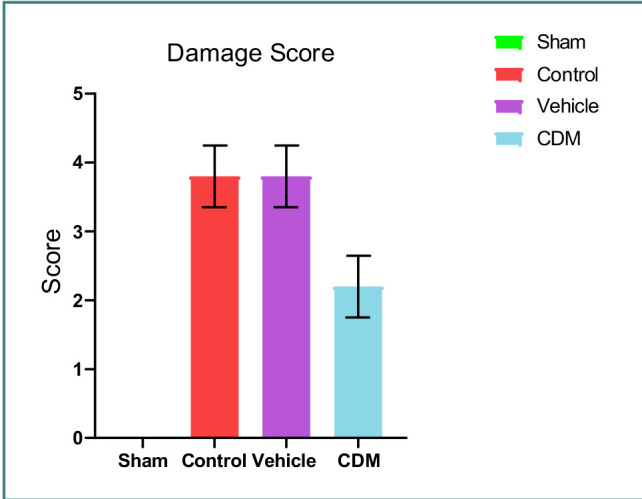
Effect of CDM on renal morphology Mean±SEM, n=5. *P≤0.05 vs sham; #P≤0.05 vs control/vehicle.

**Figure 8:**
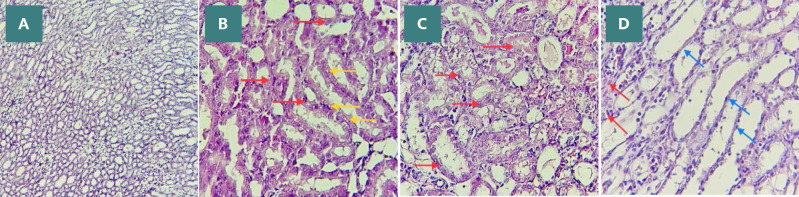
Histopathological examination of renal tissues (H&E). A: Sham group with normal renal tissue. B, C: Control and vehicle groups exhibit severe injury, including cell swelling, cytoplasmic eosinophilia (red arrows), and vacuoles (yellow arrows). D: The CDM-treated group displays mild damage with predominantly normal tubules (blue arrows).

Compared to the sham group, which exhibited normal renal tissue morphology, the tissue sections from the control and vehicle groups were characterized by severe structural damages, including loss of brush border, cell swelling, cytoplasmic eosinophilia, lumen congestion, and cast formation (Figure 7, Figure 8A-C). Renal tissue sections of rats pretreated with CDM exhibited mild interstitial congestion and tubular swelling, suggesting ameliorative influence (Figure 7, Figure 8).

## DISCUSSION

The current study demonstrated that serum levels of BUN and creatinine were significantly elevated in the control and vehicle groups compared to the sham group, which agrees with previous findings [22, 23]. This increase can be attributed to the reduced renal blood flow and glomerular filtration rate caused by ischemia. Pretreatment with CDM reduced these levels, supporting earlier studies that highlighted the protective role of CDM in renal tissues in a model of cisplatin-induced nephrotoxicity [24]. Furthermore, the levels of TNF-α and IL-1β in the renal tissues of rats in the control and vehicle groups were higher than in the sham group. This observation is consistent with earlier studies that reported increased levels of these cytokines in renal ischemia models, leading to harmful effects [25, 26]. The increase in these cytokines was likely due to hypoxia and a reduction in renal blood flow, which in turn triggers an influx of inflammatory cells into the damaged tissue [27]. Pretreatment with CDM reduced the levels of TNF-α and IL-1β in the renal tissues, suggesting its anti-inflammatory impacts. This aligns with previous findings on the anti-inflammatory effects of CDM in models of hepatic and cerebral ischemia-reperfusion injury [28, 29].

Furthermore, our study revealed that CDM reduced the levels of F2-isoprostane in renal tissues compared to the control and vehicle groups, potentially due to antioxidant properties. CDM has recently been reported to decrease pressure overload-induced cardiac dysfunction by reducing oxidative stress. Moreover, the beneficial effects of CDM in reducing and ameliorating oxidative stress may be associated with the significant reduction of iNOS expression by reducing p65/nuclear factor-B nuclear translocation and impairing nuclear factor kappa B (IB) phosphorylation and the significant elevation of eNOS expression and NO level [30]. Moreover, the current study showed a significant decrease in the levels of caspase-3 in the CDM-treated group compared to the renal IRI group. This supports previous findings that CDM has antiapoptotic effects, characterized by reduced caspase-3 expression and a lower Bax/Bcl-2 ratio [31].

## CONCLUSION

The findings of this study revealed that CDM improved kidney function by lowering serum BUN and creatinine, reducing inflammatory and oxidative markers, and demonstrating antiapoptotic properties, suggesting its therapeutic potential in renal health.
